# Flucytosine and its clinical usage

**DOI:** 10.1177/20499361231161387

**Published:** 2023-04-05

**Authors:** L. Shamithra M. Sigera, David W. Denning

**Affiliations:** Division of Infection, Immunity & Respiratory Medicine, School of Biological Sciences, Faculty of Biology, Medicine and Health, The University of Manchester, Manchester M13 9PL, UK; Division of Infection, Immunity & Respiratory Medicine, School of Biological Sciences, Faculty of Biology, Medicine and Health, The University of Manchester, Manchester, UK

**Keywords:** antifungal resistance, *Candida* endophthalmitis, *Candida* meningitis, *Candida* species, *Candida* urinary tract infection, combination therapy, *Cryptococcus species*, dematiaceous fungi, flucytosine, fungal endocarditis

## Abstract

Flucytosine is an antifungal agent first licensed in the 1970’s. However, its clinical value has long been overlooked and its availability across the globe is limited. This review highlights the important clinical and pharmacological aspects of flucytosine. This a narrative review of the clinical and *in vitro* susceptibility literature, with a focus on clinical uses for flucytosine. Detailed literature review including early literature related to primary and acquired resistance to flucytosine. Flucytosine has good antifungal activity against *Cryptococcus* species, *Candida* species, and dematiaceous fungi. Its water solubility enables good penetration into the eye, urinary tract, central nervous system (CNS), cardiac vegetations and fungal biofilms. In combination with amphotericin B, it shows early fungicidal activity against *Cryptococcus* species, and this translates to ~20% improved survival in cryptococcal meningitis. Combination therapy also reduces the mortality of *Candida* meningitis, and should be used in neonatal candidiasis because of the high frequency of CNS infection. Monotherapy for urinary candidiasis is under-studied, but is usually effective. It is probably valuable in the treatment of *Candida* endocarditis and endophthalmitis: there are few data. It is not effective for aspergillosis or mucormycosis. Flucytosine monotherapy of urinary candidiasis resulted in 22% developing resistance on therapy and failing therapy, and in 29% of 21 patients with cryptococcosis. Certain regions of the world still do not have access to flucytosine compromising the management of certain severe fungal infections. Flucytosine has an important role in combination therapy for yeast and dematiaceous infections and probably as monotherapy for urinary candidiasis, with a modest risk of resistance emergence. Facilitating access to flucytosine in those regions (especially low-income countries) might alleviate the mortality of invasive fungal diseases

## Introduction

Expansion of the susceptible patient population for fungal infections has increased the demand for antifungals, yet their availability is far from uniform across the world. Meantime, the development of new antifungals proceeds at pace, but older antifungals remain in pole positions in the market. One old antifungal is flucytosine (also known as 5-fluorocytosine or 5FC), a nucleoside analogue, which was initially used to treat tumors.^1^

**Figure 1.**
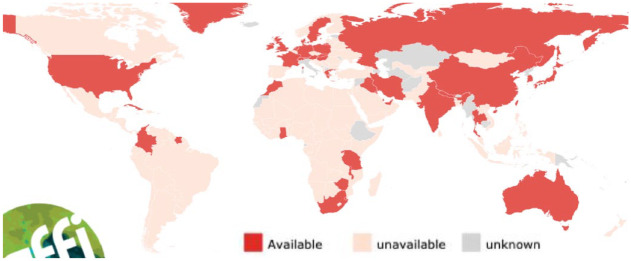
2022 country registration of flucytosine.^4^

In 1963, its antifungal effect was identified, and clinical studies began in 1968^2^ and it was initially licensed by the US Food and Drug Administration in 1974. Unfortunately, this drug is still not available in certain parts of the world. Kneale *et al.*^3^ showed that 5FC was not licensed in 71.2% of countries and is unavailable in 76.0% of countries in 2016 in their mapping of antifungal drugs in countries with more than 1 million population. The current global availability is presented in the map given in Figure 1 (see https://gaffi.org/antifungal-drug-maps/).

## Mechanism of action

5FC, a synthetic fluorinated analogue of cytosine, has no antifungal activity by itself.^5^ Each fungal cell imports 5FC through an energy-dependent process where the cytosine permease enzyme plays a vital role.^2^ Then, 5FC is converted into its active form, 5-fluorouracil (5FU), through rapid deamination by cytosine deaminase enzyme.^6^ 5FU is then metabolized into 5-fluorouridine triphosphate, which is incorporated into fungal RNA and replaces uridylic acid in fungal RNA thereby inhibiting protein synthesis.^6^ In addition, the conversion of 5-FU into fluoro-deoxyuridine monophosphate blocks the action of thymidylate synthetase resulting in inhibition of DNA synthesis.^5^ Both these mechanisms are responsible for its antifungal activity.

**Figure 2.**
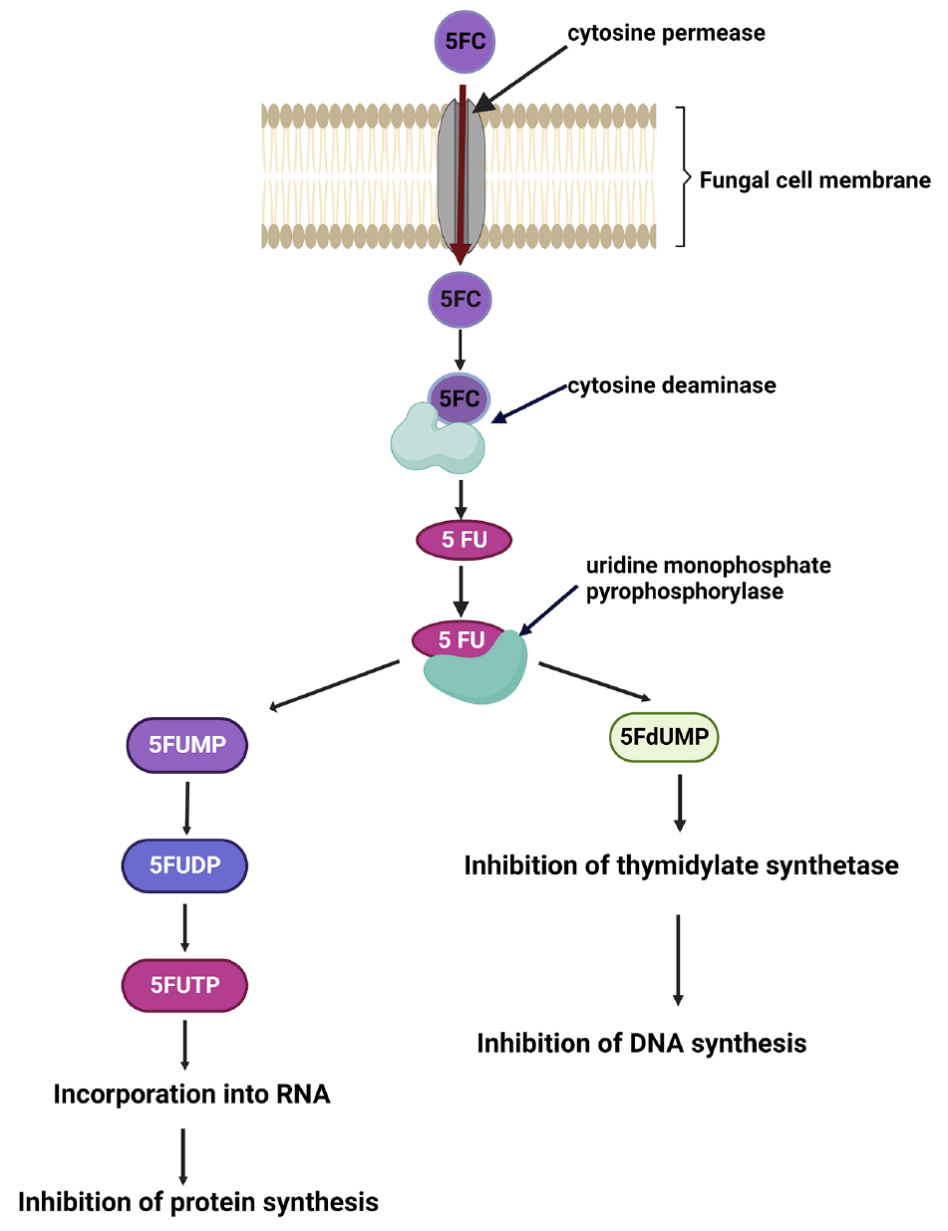
Diagram shows the mode of action of 5FC. Created with BioRender.com.

Unlike prokaryotic and fungal cells, human cells do not possess the cytosine deaminase enzyme which converts 5FC into the active form, and so, 5FC is safe in humans at pharmacological doses.^7^ However, adverse effects do occur when exposure exceeds 100 µg/ml in plasma or if it accumulates with renal dysfunction. This toxicity is explained by the discovery that the microflora of the human gut converts 5FC into 5-FU (Figure 2).^7^

5-FC is transported into the cell through an energy-dependent process by cytosine permease enzyme. Then, 5-FC is deaminated to 5-fluoraucil (5-FU) by the enzyme cytosine deaminase.

## The spectrum of activity

This drug shows a limited spectrum of activity which encompasses yeast species including *Candida* species, *Cryptococcus* species, and certain molds including dematiaceous molds but not *Aspergillus* species, any Mucorales, or endemic primary pathogens. The spectrum of activity of flucytosine is given in Table 1.

**Table 1.** Spectrum of activity of 5FC.

Fungal species	Susceptibility to 5FC
*Candida albicans*	+ +
*Candida glabrata*	+ +
*Candida parapsilosis*	+ +
*Candida tropicalis*	+ +
*Candida lusitaniae*	+ +
*Candida auris*	+
*Candida krusei*	+
*Cryptococcus neoformans*	+ +
*Cladophialophora bantiana*	+
*Exophiala* spp.	+
*Phialophora* spp.	+
*Fonsecaea pedrosoii*	+
*Fusarium* spp.	−
*Aspergillus fumigatus*	−
Mucorales	−
*Scedosporium* spp.	−
*Blastomyces* spp.	−
*Coccidioides* spp.	−
*Histoplasma capsulatum*	−

+ +: Active (usually active clinically); +: Active (maybe less active clinically); −: No activity.

## Pharmacokinetics and dosing

5FC is available in both oral and IV formulations.^8^ One of the drawbacks is the limitation of IV formulation in certain regions of the world.^1^ The use of IV 5FC which contains 35 mmol sodium chloride per 2.5 g ameliorates the nephrotoxicity of amphotericin B.^9^

**Figure 3.**
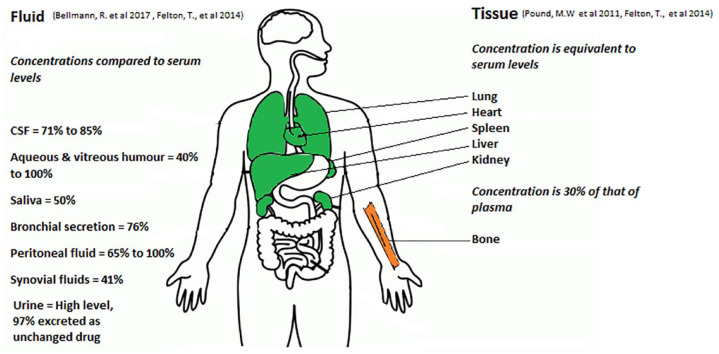
Tissue penetration of flucytosine.

Following oral administration, the bioavailability of 5FC is 76–89%. Some studies suggest that the bioavailability could be lower among late-stage HIV patients.^8^ 5FC shows little (2–4%) protein binding, and this explains the high level of circulating free 5FC levels.^10^ Owing to its high water-solubility it does not penetrate into adipose tissue and its volume of distribution ranges between 0.6 L/Kg to 0.9 L/Kg but it reaches good levels in urine, vitreous fluid, and cerebrospinal fluid (CSF).^10^ For example, the concentration of 5FC in human CSF is approximately 71–85% of that in serum.^11^ 5FC levels in saliva and bronchial secretions are 50% and 76% of those of serum, respectively.^11^ Moreover, 5FC concentrations in human peritoneal fluid are around 65–100% of those in serum.^12^ The reported concentration of 5FC in bone was 30% of that of plasma and in synovial fluids was 41%.^12^ Studies have detected approximately 40–100%, respectively, of serum concentrations of 5FC in both the aqueous and vitreous humor of human eyes.^12^ 5FC levels have been investigated in an animal model: The concentration of 5FC in the heart, liver, and spleen of a rat was similar to its corresponding serum concentration.^12^ The ratio of skeletal muscle/blood concentration in rats, who were given 5FC, was 1.1.^12^Figure 3 shows the tissue penetration of flucytosine.

5FC is mainly excreted in urine as unchanged drug (97%), and its clearance is proportional to creatinine clearance.^12^ If the patient’s renal function is normal, Cmax is reached within 1–2 hours in steady-state and the half-life of 5FC with normal renal function is 3–4 hours. Exposure increases among those with renal impairment^13^ and drug dosage intervals should be adjusted.^11,14^Table 2 shows adjustment of 5FC dosing based on renal dysfunction.^15^

**Table 2.** 5FC dosage adjustment with renal dysfunction.

GFR (ml/min) and 5FC dose adjustment		
20–40	10–20	Less than 10
50 mg/kg 12 hourly	50 mg/kg 24 hourly	50 mg/kg then dose according to levels. Dose of 0.5–1 g daily is usually adequate

5FC, 5-fluorocytosine; GFR, Glomerular filtration rate.

Elimination of 5FC during continuous renal replacement therapy and hemodialysis is anticipated owing to its small molecular weight and low protein binding nature.^11^Table 3 shows 5FC dosing with renal replacement therapy.

**Table 3.** 5-FC dosing with renal replacement therapy.

Dialysis method	Adjustments
Automated peritoneal dialysis (APD)/continuous ambulatory peritoneal dialysis (CAPD).	Give 50 mg/kg daily in 4 divided doses. Monitor levels.
Intermittent hemodialysis (HD)	Dose as in GFR < 10 ml/min, given postdialysis. Monitor trough-level predialysis, and reduce postdialysis dose accordingly
Intermittent hemodiafiltration (HDF)/High flux.	Dose as in GFR < 10 ml/min, given postdialysis. Monitor trough-level predialysis, and reduce postdialysis dose accordingly.
^a^Continuous arteriovenous hemofiltration (CAV)/continuous venovenous hemodialysis (VVHD).	Give dose as in GFR = 10–20 ml/min and monitor blood levels, predose.

5-FC, 5-fluorocytosine; GFR, Glomerular filtration rate.

a The optimal dosage for patients with renal failure requiring continuous venovenous hemofiltration or continuous venovenous hemodialysis is not yet established.^15^ Serum concentrations need monitoring.

## Side effects

5FC has a well-known side effect profile that includes both dose-related drug toxicity and idiosyncratic drug toxicity. A significant degree of dose-related toxicity such as bone marrow suppression (leukopenia and thrombocytopenia), hepatotoxicity, gastrointestinal intolerance, and renal impairment was observed during past clinical trials that used a high dose (150 mg/kg/day) of 5FC.^16^ According to clinical trials, sustained elevation of 5FC concentration greater than 100 µg/ml was associated with bone marrow toxicity and liver toxicity.^17^ However, the relatively low incidence of (4.4%) grade IV neutropenia was observed in a pooled review of seven recent drug trials which used a dose of 100 mg/kg/day.^18^ Similarly, a substudy from one clinical trial observed no significant bone marrow toxicity or hepatotoxicity following an oral dose of 100 mg/kg/day.^8^

Occasional patients may manifest idiosyncratic side effects such as skin rashes or mucosal lesions. For example, idiosyncratic reactions against 5FC were observed among dogs who developed cutaneous or mucocutaneous eruptions. Those lesions completely disappeared after discontinuation of the drug.^19^ Nausea (and vomiting) is relatively frequent.

## Therapeutic drug monitoring (TDM)

Safe and effective use of certain antifungals requires TDM which is indicated in some patients taking 5FC therapy, notably those with renal impairment and premature neonates. The requirement of TDM for 5FC is primarily dependent on its toxicity profile associated with high drug levels. In addition, evidence of variable 5FC concentrations and the emergence of drug resistance at low exposures highlights the value of TDM for 5FC.^20^Table 4 shows the commonest drugs that interact with 5FC.

**Table 4.** Drugs interacting with flucytosine.

Drug	Effect	References
Amphotericin B	Increase renal toxicity	Ashley and Dunleavy^15^
Cytarabine	The concentration of 5-FC is possibly reduced	Ashley and Dunleavy^15^
Aluminum hydroxide /magnesium hydroxide	Delays the absorption of 5-FC	Vermes *et al.*^5^
Zidovudine	Possibility increased myelosuppression	Vermes *et al.*^5^

5-FC, 5-fluorocytosine.

A few indications for TDM in 5FC have been published. 5FC TDM should be conducted at 48–72 hours after commencement of therapy, after dose adjustment, when interacting drugs start or stop or when there is uncertainty about compliance, and if there are potential clinical or laboratory manifestations of toxicity.^20^

When monitoring Cmax, sampling 2 hours after the dose is ideal and the target serum/plasma concentration should be < 100 µg/ml.^21^ The PK of Cmax greater than 100 µg/ml is frequently accompanied by hepatotoxicity and bone marrow suppression, and thus, monitoring of Cmax is mandatory in TDM.^20^ A 5FC Cmax less than 25 µg/ml is subtherapeutic and associated with the emergence antifungal resistance.^20,21^ When monitoring Cmin (to decide on dosing interval in renal dysfunction), sampling should be done just before the next dose and the target value (C min) should be greater than or equal to 25 µg/ml.^21^

However, in the absence of TDM in resource-limited settings, recommended doses of 5FC (100 mg/Kg) may be given safely along with certain basic laboratory tests (blood count and creatinine) using a dosing nomogram, if renal function is poor or changes.^22^ TDM is not required among most patients with HIV,^22^ the major exception being those with renal dysfunction.

## Flucytosine resistance *in vitro*

The degree of primary 5FC resistance varies in different fungal species. For example, the resistance rates among *C. albicans*, *C. neoformans*, and *Candida* non-albicans are reported in 7–8%, 1–2%, and 22% isolates, respectively.^5^ Resistance to 5FC is a concern in *C. auris*, an emerging multidrug-resistant non-*albicans Candida* species. Chowdhary *et al.* showed that 47% of 15 *C. auris* isolates were resistant to 5FC (another study described 60% of MIC.^23^ 5FC resistance among 10 ⩾ 64 μg/ml) when tested by the CLSI broth microdilution method. The MIC values range from 0.25 to 64 µg/ml. *C. auris* isolates with high MIC values (MIC > 32 µG/ml).^23^

In the United States, an unexplained decrease in the prevalence of resistance to flucytosine was observed in the late 1990s.^24^ Authors suggest that flucytosine might warrant being used more in treatment if the true rate of resistance is well below 10%.^24^

A few resistance mechanisms have been described related to the mode of action of 5FC within the fungal cell. Mutations of FCY1, FCY2, and FUR1 genes that encode the cytosine deaminase, purine-cytosine permease, and uracil phosphoribosyltransferase, are reported to result in resistance to flucytosine in fungi.^25^ Around 10% of 5FC resistance of *C. albicans* is related to decreased 5FC uptake by deficient cytosine permease enzyme, an enzyme that uptake 5FC into the fungal cell, due to the mutation of the FCY2 gene.^5,26–28^

Point mutation in the FCY1 gene resulting alterations in enzyme cytosine deaminase limits the conversion of 5FC to 5-FU.^26,27^ This mechanism of resistance has been observed among *C. gattii* clinical isolates.^29^ Another documented mechanism of resistance is an imitation of conversion of 5-5FC to 5-fluorouridine monophosphate (5-FUMP) due to deficiency of enzyme uracil phosphoribosyltransferase, an enzyme encoded by FUR1 genes. This is related to the mutation of FUR1 genes.^5,26,27^ A missense mutation leading to substitution of F211l amino acid of FUR1 gene has demonstrated 5FC resistance in a *C. auris* strain but remains to be confirmed as the cause of resistance.^30^ Resistance strains of *C. auris* against 5FC, especially with a missense mutation of FUR1, have also been observed and this will limit its use against *C. auris*.^31^ Phylogenomic analysis of *C. auris* isolates of the UK outbreak showed a single isolate with 5FC resistance due to substitution of phenylalanine to isoleucine FUR1 gene.^31^ In addition to the above-discussed mechanisms, increased synthesis of pyrimidine which competes with fluorinated anti-metabolites is another 5FC resistance mechanism.^5^

The rapid development of resistance during monotherapy of 5FC has long been discussed and documented.^2,32^ Secondary resistance to flucytosine emerge while on therapy for Candida infections and for cryptococcal meningitis.^33^ Chang *et al.*^25^ showed the development of a high rate of flucytosine resistance in *Cryptococcus* species at flucytosine concentrations well above its minimal inhibitory concentration. The authors presume that flucytosine could be mutagenic owing to the high degree of resistant mutants identified in their study.^25^ However, the molecular mechanisms underlying the secondary resistance are not well delineated^33^; segregation of the heterozygous state in *C. albicans* allowed expression of the presumptive resistance gene in one study.^34^ Emergence of resistance is less common in *C. glabrata*, which is haploid.^35^ In a clinical study of urinary candidiasis treated with only 5FC in 27 patients, 78% responded and secondary resistance emerged in six patients (22%) who responded poorly.^36^ One patient with *Candida parapsilosis* endocarditis treated only with 5FC, developed resistance after 37 days of therapy, documented to be attributable to an almost complete lack of cytosine deaminase activity.^37^ In a series of 21 patients with cryptococcosis given 5-FC, emergence of resistant cryptococci was linked to treatment failure in six patients (29%).^38^

## Reason for combination therapy

The concern about drug resistance occurring during therapy has led to recommendations to use 5FC in combination with other antifungal agents.^32,39^ The frequency of 5FC resistance is reduced if 5FC and amphotericin B are used combination therapy This combination was tested against *Candida albicans* strains *in vivo* using strains with various degrees of 5FC resistance; a considerable decline in 5-FC-resistant mutants was observed if combined antimicrobial therapy was used, compared with 5FC monotherapy.^40^ Consequently, it is frequently used as a combined treatment with other antifungals such as amphotericin B.^39^

Some *in vitro* studies found an additive effect of 5FC when combined with amphotericin B against *Cryptococcus neoformans*.^39^ However, results of *in vitro* sensitivity testing of the combination of 5FC with fluconazole, itraconazole B, and amphotericin B against *Cryptococcus* species show much variation but most studies have not observed antagonism.^41–43^ In addition, a study observed that there is no antagonism of combination therapy of flucytosine and amphotericin B, micafungin, or voriconazole against *Candida auris*.^39^ Moreover, an *in vitro* study of combination therapy of flucytosine with amphotericin B against *Exophiala spinifera* observed an additive effect without antagonism.^44^ The effectiveness of combination therapy has been evaluated *in vivo* studies with variable results.^45^ A synergistic or additive effect of combined amphotericin B and 5FC was observed in mouse models with candidiasis and cryptococcosis.^45^ However, the same study detected only indifferent or mild additive effects against aspergillosis in mouse models,^45^ consistent with poor efficacy of 5FC for aspergillosis.^46^ Combination therapy with 5FC and amphotericin B was efficacious in a localized candidiasis mouse model showing either an additive effect or even a synergistic effect.^40^ Efficacy for CNS phaeohyphomycosis has also been observed in a mouse model.^47^

However, in treating fluconazole-resistant urine or ocular infections, 5FC may be tried as monotherapy, as responses do occur, and resistance emergence is not inevitable. In addition, 5FC has been evaluated as monotherapy in the treatment of cryptococcal meningitis with promising results and development of resistance was not observed in one animal study.^32^

## Clinical uses of flucytosine

### Cryptococcal meningitis

Cryptococcal meningitis is more prevalent among at-risk groups including patients with HIV, on cell-mediated immunity suppressive treatment (i.e. after transplantation) or with decompensated liver cirrhosis.^48^ This lethal disease is responsible for 15–20% of total HIV-related deaths.^49^ Cryptococcal meningitis also occurs in people with no underlying disease. However, the true fungal burden of cryptococcosis might be larger than we observe as the diagnostic facilities and reporting systems are not evenly distributed across the world.

The mortality of cryptococcal meningitis ranges from 22% to 96% at 10–12 weeks in low- and middle-income countries in comparison with 9–15% in Western Europe and North America.^50^ Use of 5FC with amphotericin B lowers mortality and reduces subsequent relapse.^51,52^

Most guidelines, including those from the World Health Organization (WHO), recommend the use of combination therapy of 5FC and amphotericin B at the induction phase of treatment with the aim of rapid clearance of the yeast form (Table 5).^53,54^

**Table 5.** Indications for flucytosine.

Indications for flucytosine
1. Cryptococcal meningitis
2. *Candida* endocarditis, *Candida* endophthalmitis, *Candida meningitis*, *Candida* symptomatic urinary tract infections caused by azole resistance *Candida* species, neonatal candidiasis (because of the high proportion with meningitis)
3. Treatment of dematiaceous fungi (phaeohyphomycosis and chromoblastomycosis)

5FC has very good tissue penetration into the central nervous system compared with amphotericin B and it is recommended as a combined therapy.^55^ A trial evaluating the treatment of cryptococcal meningoencephalitis among AIDS patients found that CSF culture negativity is 9% more frequent among patients with amphotericin B plus 5FC compared with amphotericin B monotherapy at 2 weeks of therapy.^17^ Moreover, a randomized trial conducted in Thailand evaluating four different antifungal therapies among patients with AIDS with the first episode of cryptococcal meningoencephalitis revealed that the most rapid fungicidal regime was amphotericin B plus 5FC.^56^ However, cryptococcal induction therapy is not limited to the combination of amphotericin and 5FC, and here 5FC can also be used in combination with fluconazole. For example, the ACTA study revealed that 1 week of combined therapy of 5FC and amphotericin B and 2 weeks of combined therapy of 5FC and fluconazole, both are effective in induction therapy for cryptococcal meningitis.^52^

### *Candida* and flucytosine

The use of 5FC in the treatment of *Candida* infections is more frequent in the setting of endocarditis, endophthalmitis, meningitis, and symptomatic urinary tract infections caused by azole resistance *Candida* species.^57^ In these circumstances, it is usually combined with amphotericin B or other antifungals with the intention of avoiding the emergence of antifungal resistance.^57^

### *Candida* endophthalmitis

Flucytosine concentrates in the eye and its use in fungal eye infection has been highlighted (Table 5).^58^ In ocular candidiasis, the combination of liposomal amphotericin B and 5FC is advised in guidelines especially if the susceptibility of causative *Candida* species is unknown.^59^ The high molecular weight and high protein-bound nature of amphotericin B limit its penetration through the blood ocular barrier.^60^ Consequently, its penetration into the posterior chamber is suboptimal precluding its activity in *Candida* endophthalmitis.^57^ In contrast, 5FC reaches approximately 100% of serum concentrations of 5FC in vitreous humor of human eyes.^12^

### *Candida* urinary tract infection

Fungal urinary tract infections attributable to *Candida* spp. are common in hospitalized patients and those with indwelling catheters.^61^*Candida* spp. have been observed to be responsible for 10–30% of hospital-acquired urinary tract infections.^62^*C. albicans* is the most prevalent *Candida* species isolated from urine specimens, reaching 50–70%.^61^ However, urinary tract infections (UTIs) with C*andida* non-*albicans* species, with high resistance profiles, are becoming more frequent.^62^ For example, *C. glabrata* and *C. tropicalis* have been reported as causative agents for UTIs with a value of 10–35% of cases.^61^*C. glabrata* is frequently reported among patients with hematological malignancies and renal transplant recipients.^61^ In addition, *C. parapsilosis* and *C. krusei* are responsible for 1–7% and 1–2% of cases, respectively.^61^

Although fluconazole is frequently used as the antimicrobial against *Candida* UTI, its value in treatment is limited for resistance isolates including *C. glabrata, C. krusei, and C. auris*.^62^ In addition, lipid formulations of amphotericin B with broad-spectrum activity do not reach an adequate level in urine and have no value in the treatment of UTI.^62^ In these circumstances, 5FC can be used effectively (except for *C. krusei*).^62^ 5FC is secreted in its active form in urine and reaches high concentrations.

5FC is a useful treatment option in refractory *Candida* UTI and especially when due to azole-resistant *Candida* species such as *C. glabrata* and should be considered with *C. auris* (Table 5).^61,63^

### Neonatal candidiasis

About 40% of neonatal candidiasis involves the CNS, a much higher proportion than in adults. A prospective study evaluating the benefits of adding 5FC to amphotericin in the setting of neonatal candidiasis showed that the median time of clearance of CSF was faster among patients who received amphotericin deoxycholate alone compared with combined therapy of amphotericin B deoxycholate and 5FC.^64^ A combination of amphotericin B and 5FC is commonly used for neonatal CNS candidiasis cases.^57^

### *Candida* meningitis

CNS fungal infection rates are rising at-risk population, USA hospital discharge data from 2011 to 2014 showed that fungal etiologies accounted for 2.7% of cases of meningoencephalitis.^65^ CNS fungal infections are due to an array of fungal species including *Candida, Cryptococcus, Aspergillus, Coccidioides, and Histoplasma capsulatum* and even rarer cases of phaeohyphomycosis and sporotrichosis.^65^

*C. albicans* is still the most prevalent species causing *Candida* meningitis; however, the rate of detection of non-*albicans Candida* species is gradually rising.^65^ Although *Candida* meningitis is an uncommon condition, it is associated with poor outcomes mandating urgent and appropriate antifungal therapy.^59^

Systemic administration of liposomal amphotericin B with or without 5FC is recommended in guidelines (Table 5).^59^ Casado *et al.*^66^ analyzed the treatment of a group of HIV patients with *Candida* meningitis and found that patients managed with a combination therapy of 5FC and amphotericin B had a 22% better survival, mortality falling from 33% with amphotericin B alone to 11% when the same cohort was treated with combined therapy of amphotericin B and 5FC.

### Vaginal candidiasis

Treatment of vaginal candidiasis due to *C. glabrata* is a challenge to the physician because it usually fails azole therapy.^67^ White *et al.*^68^ described three patients with recurrent vaginal candidiasis due to *C. glabrata* who were successfully treated with combination therapy of flucytosine and amphotericin in lubricating jelly. In addition, complicated vaginal candidiasis due to *C. glabrata* irrespective of boric acid and azole therapy has been successfully managed with topical flucytosine vaginal cream with no or minimal side effects.^67^ Unfortunately, topical vaginal cream is not widely available in the market.^67^

### Role in fungal infective endocarditis

Although fungal endocarditis is an uncommon disease accounting for 2–4% of all endocarditis, it carries a very high mortality rate of 30–50%, demanding the prompt commencing of the best course of antifungal treatment.^69^

The rising incidence of fungal endocarditis is attributed to expanding number of at-risk populations such as intravenous drug users, previous cardiac surgeries, prosthetic valves, prolonged parenteral nutrition, prolonged use of broad-spectrum antibiotics, and patients with immunosuppressive therapy.^69^

*C. albicans*, *Candida* non-*albicans* species with a high degree of antifungal resistance, and *Aspergillus* species are each responsible for 25% of fungal endocarditis cases. The remaining 25% of fungal endocarditis cases are caused by many rarer fungi.^70^

In treatment guidelines, a high dose of echinocandin or amphotericin B with or without 5FC is recommended in *Candida* endocarditis as the initial therapy along with valve replacement (Table 5).^71^ However, penetration of amphotericin B inside the vegetation is insufficient, and this obstacle is overcome by combination therapy with 5FC.^72^ Patients who are not a candidate for valve replacement show more favorable outcomes when treated with a combination therapy of amphotericin B and 5FC over amphotericin alone.^71^

In addition, patients with infective endocarditis due to *C. parapsilosis, Candida famata*, and *Candida guilliermondii* should be preferably treated with amphotericin B and 5FC as these species show less susceptibility to echinocandins.^71^

### Treatment of *C. auris* infections

*C. auris* is a multidrug-resistant emerging yeast that has been highlighted due to its close association with severe nosocomial outbreaks.^73,74^ It has been isolated in over 35 countries on five continents across the world.^74^ It is characterized by a high degree of transmissibility which is explained by its ability to persist on surfaces for a long duration and its ability to colonize skin and body sites.^74^ Moreover, its resilient nature is described by its nature of high osmotolerance (more than 10% NaCl) and high thermotolerance (more than 40°C), and its ability to survive exposure to many disinfectants.^74^ Infections due to *C. auris* are associated with a high degree of mortality which varies across the different studies; in some from 28% to 66%.^74,75^ Another study reports that *C. auris* associated mortality rates expanded from 28% to 56%.^76^ The estimated crude in-hospital mortality rate of *C. auris* candidemia ranges from 30% to 72%.^77^ In addition, an extremely high mortality rate of more than 83% was observed among co-infection of *C. auris* and COVID-19 infection in Mexico.^74^

This high degree of mortality rate may be attributed to its high antifungal resistant profile.^73^ Studies from different countries reported different degrees of resistance to different antifungals against *C. auris*.^77^ Around 90%, 30% and < 5% of *C. auris* isolates in the USA showed resistance to fluconazole, amphotericin B, and echinocandin respectively based on current CDC breakpoints.^74^

Echinocandins are recommended as the initial therapy for *C. auris* infections among adult patients. Both primary resistance to echinocandin and emergence while on therapy is a growing concern.^74^ In refractory cases, 5FC would be a good partner in a combination, especially in urinary tract infections due to *C. auris*.^63^ Studies of drug combination therapy by checkerboard assay found an indifferent interaction between 5FC and other antifungals such as amphotericin B, micafungin, and voriconazole toward *C. auris* isolates.^39^ Consequently, 5FC seems to be an attractive option in combined therapy for the treatment of infections due to *C. auris*.

However, certain studies were not supportive of the use of 5FC in combination therapy with amphotericin in the treatment of refractory cases of *C. auris* infections based on the combined side effects profile.^78^ Resistance strains of *C. auris* against 5FC, especially with a missense mutation of FUR1, have also been observed and this will limit its use against *C. auris*.^31,77^

### Treatment of dematiaceous fungi

5FC shows good *in vitro* activity against many dematiaceous molds, a group of fungi that includes melanized, saprophytic fungi such as *Alternaria* spp., *Cladophialophora bantiana*, *Exophiala dermatitidis*, *Phialophora* spp., *Fonsecaea pedrosoii*, and *Rhinocladiella mackenziei*.^79^ These fungi usually result in mild, superficial cutaneous infections such as localized cutaneous and subcutaneous nodules, eumycetoma, keratitis, fungal sinusitis or just colonize the respiratory tract.^79,80^ Although mild cases can be managed with surgery or azole therapy alone, more refractory cases need enhanced therapy. Successfully treated cases have been reported with the combination therapy of new azoles and 5FC.^79,80^

Infrequently, these melanized molds also result in deep-seated invasive infections such as cerebral phaeohyphomycosis and disseminated infection among at-risk populations where the prompt start of effective drug combination is essential.^81,80^ In these circumstances, 5FC plays an important role in combination therapy, especially if the patient is not a candidate for surgery. In these circumstances, combined therapy of triazole, echinocandin, and 5FC is recommended as the first-line therapy (Table 5).^79,80^

### Activity against *Aspergillus*

The world sees over 300,000 patients with invasive aspergillosis annually,^81,82^ with a high mortality rate which is at least 28.5% when diagnosed and with current treatment.^83^ The picture is more complicated by the emerging azole resistance among *Aspergillus* spp., especially for voriconazole, the drug which is widely recommended by numerous treatment guidelines.^82^ Combination antifungal therapy is practiced in refractory cases of invasive aspergillosis.

The combined therapy of amphotericin B and 5FC was popular in the treatment of CNS aspergillosis before voriconazole became available.^21^ It appears a good option for the treatment of CNS aspergillosis because 5FC reaches therapeutic levels in CSF after systemic administration.^84^ In addition, 5FC in combination with amphotericin B has been used to treat *Aspergillus* endocarditis and complicated *Aspergillus* urinary tract infections.^85,86^

However, there has been controversy regarding the effectiveness of 5FC against *Aspergillus* species because the activity of 5FC on *Aspergillu*s species *in vitro* is dependent on the pH.^87^ Studies observed high MIC values of 5FC against *A. fumigatus* at pH 7 compared with low MIC values obtained when investigated at pH 5.^83^ A small randomized study of 5FC combination therapy with or without amphotericin B in hematology patients with invasive aspergillosis showed no benefit or even hint of benefit.^46^ The use of 5FC for aspergillosis is probably ineffective.

## Research gaps and future directions

The use of topical 5FC in refractory vaginal candidiasis has been appreciated for two decades.^67,68^ Although topical flucytosine is not widely available in the market, this option could be extended to other refractory mucocutaneous *Candida* infections, including those associated with primary immunodeficiency (IL-17 deficiency and chronic mucocutaneous candidiasis) and HIV patients. Clinical trials are necessary.

There are inadequate evaluations of monotherapy for urinary candidiasis, especially if fluconazole-resistant. The apparent emergence of resistance at a 25% rate, given the general lack of antifungal agents that get into the urine, does not seem excessive, and is certainly not a general public health threat.

There are conflicting data on the use of flucytosine for disseminated candidiasis in premature neonates. Addition work on this topic is warranted, given the severity of these infections. Combination with fluconazole or micafungin should be studied, as well as amphotericin B.

While some strains of *C. auris* are resistant to flucytosine, not all are, and some strains are not only resistant to fluconazole but also amphotericin B and/or echinocandins. Nothing is known about the emergence of resistance to flucytosine during therapy, as monotherapy or in combination.

For some patients, administration of flucytosine 4 times a day is difficult to sustain, especially of therapy is prolonged and out of hospital. Sustained release formulations allowing twice (or 3 times) daily would be advantageous.

## Conclusion

5FC is an antifungal that acts against many fungi. Combination therapy with other antifungals is recommended in refractory cases including treatment of infections with resistant fungi, and fungal infections associated with biofilm and vegetation formation. Its inherent features of low molecular weight, less protein binding, and high water solubility facilitate its penetration into deep tissues which highlights its use in the treatment in severe fungal infections of the eye, urinary tract, CNS, and fungal infections associated with vegetations and biofilms. Improvement of accessibility to 5FC in resource-limited countries will mitigate the mortality invasive fungal diseases.

## Declarations

**Consent for publication:** Not applicable.

**Ethics approval and consent to participate:** Not applicable.

**Availability of data and materials:** Not applicable.
